# The role of cannabis clinics in the health system: a qualitative study of physicians’ views in New Zealand

**DOI:** 10.1186/s12913-022-09021-y

**Published:** 2023-01-04

**Authors:** Withanarachchie Vinuli, Rychert Marta, Wilkins Chris

**Affiliations:** Shore & Whāriki Research Centre, College of Health, Massey University, Auckland, New Zealand

**Keywords:** Cannabis, Cannabidiol, CBD, Medical marijuana, Medicinal cannabis, Cannabis clinic, Qualitative research

## Abstract

**Background:**

Privately-owned cannabis clinics have sprung up in many jurisdictions where medicinal cannabis has been legalised and provide an alternative pathway for patients who are unable or unwilling to access a prescription for cannabis-based medicinal products from their usual healthcare providers.

**Aims:**

This study aimed to explore physicians’ views on cannabis clinics, including their perceptions of the role cannabis clinics play in the wider health system.

**Methods:**

A qualitative study using in-depth, semi-structured interviews with thirty-one physicians affiliated with private and community clinics in New Zealand (including cannabis clinicians, GPs, and specialist doctors). The interviews were conducted from July to December 2021. Data were transcribed and analysed using inductive thematic analysis.

**Results:**

Cannabis clinicians positioned themselves as (1) “service providers”, facilitating consumer access to cannabis prescriptions and products, and (2) “educators”, providing advice to patients and the wider physician community. While general practitioners and specialists recognised the benefits of specialised cannabis clinics (i.e., knowledge of products and a non-judgmental environment), they questioned the limited evidence of clinical efficacy for cannabis, potential financial conflicts of interests of cannabis clinicians that may blur their clinical judgement, and the risk of compartmentalising patients’ healthcare.

**Conclusions:**

Our paper raises a number of challenges with attempting to integrate cannabis clinics into the wider health system.

## Introduction

The last two decades have witnessed a proliferation of regulatory regimes legalising cannabis for medicinal purposes [1]. Strong public demand for medicinal cannabis (MC) products has played a vital role in developing and shaping these policies [2]. Canada, Israel, and California were at the vanguard of MC law reform, having permitted legal access to medicinal cannabis since the 1990s through prescriptions and discretionary exemptions [3, 4]. Currently, the majority of the US states, Australia, and many European countries including the UK have enabled access to cannabis-based products with a prescription or recommendation from a physician [1]. The eligible conditions and products vary between countries, meaning clinicians’ engagement with medicinal cannabis regimes depends on the regulations implemented in their environment. In the UK, for example, only specialist registered physicians can prescribe cannabis-based products as “unlicensed medicines” within their scope of practice [5], whereas in Australia, clinicians can prescribe medicinal cannabis on an individual patient basis with state and governmental approval [1].

New Zealand (NZ) enacted its Medicinal Cannabis Scheme (MCS) in April 2020 following public pressure to provide relief to terminally ill patients. Products are assessed by the Ministry of Health against “minimum quality standards” before distribution via pharmacies or ‘dispensaries’ operated by cannabis clinics in NZ. The NZ MCS now allows any doctor to prescribe medicinal cannabis products, that meet the MCS quality standards, to any patient with a condition they deem suitable. At the time of writing, 19 cannabis products met the MCS standards, with 18 more under regulatory assessment [6]. “Approved” products are in the form of oils, sprays or dried cannabis herb products for use as a tea or for vaping (while products for smoking are not allowed). As in many countries, the NZ MCS has experienced a number of challenges in the early stages of implementation, including poor health stakeholder engagement, costly products, limited product range, and a strong illegal market presence [7–9].

A number of studies evaluating clinicians’ attitudes towards MC have found a lack of standard training and limited scientific evidence for cannabis therapies are key barriers to prescribing cannabis medications [10–13]. These doubts among clinicians run alongside the proliferation of private medicinal cannabis clinics that are providing access to cannabis products [14]. Due to the relatively new emergence of cannabis clinics outside of the United States, their role in the health system, including how they are perceived by the wider physician community, is still developing. Given that initial evaluations of the NZ MCS have identified similar problems [7], this article aims to explore how cannabis clinics function within New Zealand’s broader healthcare system.

## Background – Cannabis clinics

Cannabis clinics provide an alternative pathway for patients who are unable or would prefer not to access a prescription through their usual healthcare providers [15]. Some have described cannabis clinics as “a safe space” for cannabis naïve patients to inquire about a drug that until recently was prohibited in many jurisdictions [16]. Medicinal cannabis programs have operated in a number of jurisdictions in the United States since 1996 (following implementation of the first scheme in California) and several major states including Colorado, New Jersey, Pennsylvania, and New York facilitate access to cannabis clinics [17]. Patients access these clinics by physician-referral or self-referral through word of mouth, community out-reach and marketing. Typically, following medical assessment, a state-authorised doctor or nurse completes a medical evaluation and determine patients’ eligibility for a prescription or a ‘recommendation’ [17].

Cannabis clinics largely appear to service patients with pain, neurological, and gastroenterological issues who require long-term care [14]. A systematic review found moderate-level evidence for the use of cannabis to treat chronic pain, chemotherapy-induced nausea and vomiting, and spasticity as a result of multiple sclerosis [18]. Therefore, cannabis clinicians’ capacity to accurately match cannabis-based products to a patient’s specific condition, without supporting scientific evidence for efficacy, has been questioned [14]. A 2019 survey found that 35 cannabis clinicians in the US who had made 160,000 cannabis ‘recommendations’ to patients across their combined practicing careers self-educated by consulting various conferences and peer-reviewed medical articles [19]. This suggests without formal training, cannabis knowledge across these clinicians is unlikely to be uniform as the quality and breadth of advice they can provide is contingent on which resources they consult.

In New Zealand, Te Whatu Ora (Health New Zealand) is the government agency that manages the publicly funded health system, including hospital, specialist, primary, and community services. The latter services are purchased and delivered through the four regional sectors of Te Whatu Ora. Evidence to date suggests NZ GPs are apprehensive about recommending MC to patients [20] and only one in three patients requesting a MC prescription from their GPs, receive it [9]. Anecdotally, cannabis clinicians in NZ appear to be filling the gap left by reluctant GPs by offering specialist consultations and access to products in their private clinics. Cannabis clinics in NZ are commonly run by registered GPs with a special interest in cannabis who do not require formal training to practice within this cannabis-specific scope and can be accessed by patients privately without a referral. A recent NZ survey of 3847 medicinal cannabis users found MC was most commonly used to treat pain, mental health and substance use disorders, and sleep conditions [9]. An internal audit of patient records at a NZ cannabis clinic also found 70% of patients reported satisfaction with cannabidiol used mainly to treat non-cancer pain and mental health [21].

In December 2021 (during recruitment for this study) our web-based search found 29 physicians across nine clinics practicing cannabis therapy or advertising the prescribing of cannabis-based products in NZ. Typically located in major cities, some cannabis clinics offer telehealth services to patients around the country. In August 2022, this number has grown to approximately 40 physicians across 11 clinics. New Zealand thus provides a unique backdrop against which to explore this emerging phenomenon of cannabis clinics and their role in a national health system at an early stage of implementing the new Medicinal Cannabis Scheme.

## Method

One-on-one semi-structured interviews were conducted with 31 New Zealand clinicians who had discussed medicinal cannabis with their patients in the past 6 months. Participants included 16 general practitioners (primary care) and 15 specialist clinicians (including 7 pain specialists, 5 working in palliative care, 1 psychiatrist, 1 anesthesiologist, and 1 gynecologist). Seven of the interviewed doctors identified as “cannabis clinicians” (6 GPs and 1 specialist), i.e., they worked in a specialised cannabis clinic and/or advertised as a prescriber of cannabis-based products.

Recruitment aimed for a diverse participant group including doctors from primary and specialist care, working in public and private settings (including in cannabis clinics), who have had recent engagements with patients about medicinal cannabis. Participants were recruited throughout NZ from July to December 2021 via a research invitation mass emailed to approximately 30 primary health organisations (PHO’s) and 9 cannabis clinics across New Zealand. The research invitation was also distributed via the Royal New Zealand College of General Practitioners mailing list, the second author’s professional network, and snowball referrals. Participation in the study was voluntary and anonymity was assured to the respondents. The Massey University Human Ethics Committee provided ethics approval for the study (SOA 18/85).

All participants who responded to the recruitment email or advertising and agreed to participate in the study (i.e., provide informed consent) were interviewed, provided they fulfilled inclusion criteria (i.e., currently practising and had discussed cannabis as a treatment option with their patient(s) in the past 6 months). Four participants were interviewed in-person and twenty-seven by Zoom video conference (the change in interviewing approach was necessitated by the COVID-19 lockdown). Interview times ranged from 21 to 94 minutes, with a mean duration of 53 minutes. Thirty-one physicians were interviewed. Ten of the interviewed clinicians practiced in private clinics and twenty-one in community clinics, which are supported by mixed government and private funding. Twenty-one respondents worked in the Auckland area (the biggest city in New Zealand), seven in the Northland region, and three in the South Island of New Zealand. The number of years interviewees had practiced medicine ranged from 5 to 55 years, with a mean of 27.5 years (Table 1).

**Table 1:** Summary of sample characteristics

	N	%
Gender		
Male	18	58
Female	13	41.9
Specialty		
General practice	16	51.6
Pain	7	22.5
Palliative care	5	16.1
Psychiatry	1	3.2
Anaesthesiology	1	3.2
Gynaecology	1	3.2
Geographical location		
Auckland	21	67.7
Hamilton	3	9.6
Taranaki	1	3.2
Northland	2	6.4
Christchurch	2	6.4
Dunedin	1	3.2
Queenstown	1	3.2
Practicing clinic		
Private	10	32.2
Community	21	67.7
Seniority		
> 10 years	6	19.3
10–20 years	8	25.8
21–30 years	6	19.3
31–40 years	7	22.5
< 41 years	4	12.9

A 36-item interview schedule facilitated participant conversations and demographics collected included clinical specialty, seniority level, and practice location. Interviews included questions about general attitudes towards the use of cannabis in clinical practice, knowledge and beliefs about MC, conversations about MC with their patients, perceived barriers and facilitators to prescribing MC in New Zealand, and experiences and opinions of cannabis clinics and their role in the regime. Participants were asked to rate their knowledge of the properties of MC products and the NZMCS from a scale of 1 to 10 (from poorly to very informed). They were also asked to report how many patients had enquired about MC with them in the last 6 months and how many MC prescriptions they had issued. As the NZMCS was introduced in a phased implementation, the last 6 months captured the latest and most important development stages. In addition, based on previous survey experience, the last 6 months (rather than 12 months) enhanced physicians’ capacity to accurately recall their medicinal cannabis discussions and related outcomes. Given that the NZMCS is a new policy and for most physicians’ patient requests for medicinal cannabis will be rare and novel events, it is reasonable to assume they can accurately recall the number of patient request during the past 6 months. In the case of cannabis clinics, where clinicians may deal with large numbers of cannabis patients, the aim of question was to provide a broad accurate indication of the number of patients seen per week rather than a precise week by week count. Interviewees were encouraged to provide additional information or clarify their answers after completing of the interview i.e. consult their records, review their interview transcripts, and amend their answers if they requested it. As an open-ended question, physicians were also asked their understanding of which medical conditions that cannabis may be useful to treat i.e. “Do you believe cannabis is useful for any particular conditions or groups of patients (for example, palliative care)?”

The interviews were conducted and audio-recorded by the first author, and transcribed ad verbatim. Nvivo software was used to code the transcripts and analyse the data by first and second author to ensure investigator triangulation (codes and themes were discussed in a series of meetings by the first and second author). Saturation was achieved at the 20th interview during the inductive coding process, in which core themes were developed from the interview data through a bottom-up approach [22]. Qualitative description approach, characterized by low-inference interpretation, was used to analyse and report the data, with rich descriptions and quotes used to illustrate key findings from interviews [23, 24].

### Patient and public involvement

No patients were involved in this study. The interview guide was informed by initial findings from prior interviews with medicinal cannabis users in New Zealand (earlier stages of this project). This study is part of a larger public health study exploring implementation of the Medicinal Cannabis Scheme in New Zealand. Authors are academic health researchers with expertise in studying cannabis use, cannabis markets, and drug policy change.

## Results

### Physicians’ experiences with medicinal cannabis in clinical practice

The frequency with which the clinicians discussed medicinal cannabis with patients ranged from 2 to 350 times in the last 6 months. On average, cannabis clinicians had considerably more frequent discussions with patients seeking cannabis than GPs and specialists (172 vs. 27 vs. 32), self-rated their knowledge of medicinal cannabis more highly than community GPs and specialist doctors (non-cannabis clinicians) (7.7/10 vs. 4.5/10 vs. 4.7/10), and issued more prescriptions (150 vs. 7 vs. 4) (Table 2). Most cannabis clinicians had a background in general practice (one of the seven cannabis clinicians was a specialist). Of the 20 physicians who had prescribed medicinal cannabis in the last 6 months, the types of conditions mainly prescribed for were pain, followed by sleep conditions and mental health. Cannabis clinicians (*n* = 7) were most likely to report cannabis as effective for pain (100% of cannabis clinicians), psychiatric conditions (60%), poor sleep (30%), and epilepsy (30%). GP’s (*n* = 10) were most likely to consider cannabis appropriate for poor sleep (70% of GP’s), pain (60%), and psychiatric conditions (40%). Specialists (*n* = 14) generally only considered cannabis appropriate for pain (50% of specialists) and psychiatric conditions (40%).

**Table 2:** Knowledge of medicinal cannabis and legislative reform, and number of cannabis discussions and prescriptions written in the last 6 months (mean, range)

Medical professional	Cannabis clinicians (*n* = 7)		General practitioners (*n* = 10)		Specialists (*n* = 14)	
	Mean	Range	Mean	Range	Mean	Range
Self-rated knowledge of medicinal properties of cannabis (scale 1–10)	7.7	6–9	4.5	2–8	4.7	1–8
Self-rated knowledge of NZ’s Medicinal Cannabis reform (scale 1–10)	6.5	5–9	3.3	0–8	4	1–8
Number of discussions about cannabis as a treatment option in the last 6 months	172	30–350	27	2–100	32	2–168
Number of cannabis prescriptions written in the last 6 months	150	24–299	7	0–20	4	0–7
Estimated % of cannabis discussions that resulted in a prescription	90%		32%		10%	

The main themes identified were the role of cannabis clinics as service industries, the non-judgemental clinical setting they provide patients, their role as educators to the wider physician community, and the myriad of concerns non-cannabis clinicians have about their role in the health system, including conflicts of interest, a single treatment focus, and risks of compartmentalising health.

### Cannabis clinics as a “service industry”

Cannabis clinicians espoused a strong belief in the therapeutic benefits of cannabis. They felt they provided a specialist service that met patient demand for MC and when it was was hard for patients to access via their GPs, they offered an alternative pathway for referrals from other physicians if they *“don’t feel confident or comfortable prescribing”* (Cannabis clinician 3). They positioned themselves as a “service industry” responding to consumer demand for a particular health service and filling a void created by other physicians’ reluctance to prescribe cannabis:

*“Patients have said that smoking cannabis is the only thing that gives them relief from pain, anxiety, depression and sleep problems. I read the research and those things do respond to cannabis and I’m there for the patient and willing to prescribe.”* (Cannabis clinician 1; 250 medicinal cannabis discussions in the past 6 months, 250 prescriptions).

A number of cannabis clinicians emphasised patient-centred motivations for their involvement in cannabis prescribing. One participant recalled how they began a private cannabis clinic after having been the sole medical practitioner willing to prescribe cannabis in their area. They astutely observed that cannabis clinics would not need to exist as an intermediary service for MC prescriptions if there was greater uptake and interest by mainstream medical providers:

*“... the only reason you’ve got medicinal cannabis clinics is because we’re in a position where people are uneducated … For me, CBD and medicinal cannabis would be prescribed by all GPs as part of their normal training. Why would you need a separate clinic? The only reason I’m doing it as a separate clinic is because no one else is doing it and no one else is providing it...”* (Cannabis clinician 4, 192 medicinal cannabis discussions in the past 6 months, with the vast majority of these patients received a prescription).

Cannabis clinicians felt their patients benefited from the personalised cannabis treatment service they provided via private consultations. Three of them explicitly stressed the comprehensiveness of their consultations, including discussing patients’ full medical history with cannabis both recreationally and medically. This, they argued, informed their ability to tailor, titrate, and monitor MC prescriptions to more closely fit the individual needs of patients compared to a standard 15-minute GP appointment that might cover MC amongst other issues:

*“Well, it takes the time. I allow 40 minutes for my first patient visit. If I used a nurse, that might cut down my time but patients enjoy coming here and having the personalised attention of me taking them through the whole process, taking a history, assessing the problem, and advising on what sort of treatment might be possible and then explaining how they can take it.”* (Cannabis clinician 1; 250 medicinal cannabis discussions in the past 6 months, 250 prescriptions).

GPs and specialists not working in cannabis clinics articulated two main benefits of cannabis clinics. First, they commented that cannabis clinicians were well positioned to advise patients on cannabis therapy due to the volume of consultations they conduct and learn from in their daily practices, for example:

*“The people who are providing the service in the [cannabis] clinics will get, you know, quite adept and experienced at prescribing and being able to advise patients what to expect.”* (Specialist 10, 12 medicinal cannabis discussions in the past 6 months, 0 prescriptions).

### Cannabis clinics as non-judgemental providers

Second, some non-cannabis clinicians recognised that cannabis clinics provide a non-judgmental environment for patients to discuss cannabis as a treatment option. This addresses current access barriers to prescriptions faced by patients, including social stigma, physicians’ reluctance to prescribe, and patients’ fear of judgement:

*“There is a place definitely if patients are too embarrassed or shy to ask their GP or they think they’ll be discriminated or reacted against about it or if they’ve asked the GP and the GP has said no. There is still a place [for cannabis clinics].”* (GP 7, 20 medicinal cannabis discussions in the past 6 months, 15 prescriptions).

Cannabis clinicians elaborated on the above sentiment and discussed strategies they used to create an open-minded setting for patients, or “clients”, to share their history with cannabis for therapeutic and recreational purposes. They facilitated this environment from the initial consultation to build rapport with patients and de-stigmatise cannabis use as a medical treatment:

*“I’m here to help them, not criticise them. I get that over with as best as I can, but then also I go into the whole quick bird’s eye view of the endocannabinoid system and how it works... so often people say oh that’s a huge relief, I’m really thankful that you told me all of that.”* (Cannabis clinician 4, 192 medicinal cannabis discussions in the past 6 months, with the vast majority of these patients receiving a prescription).

### Cannabis clinicians as educators

The interviews with cannabis clinicians revealed that most patients self-referred to cannabis clinics, with GP and specialist referrals being less common. Despite this trend, some cannabis clinicians expressed an interest in educating GPs who were apprehensive about providing a MC prescription, concerned about the risks of adverse effects, or misinformed about the legality of prescribing:

*“Clinics like ourselves are motivated to get the word out and that’s why we’re doing some education for doctors... to try and break down some of these assumptions that medicinal cannabis is too tricky or it’s too dangerous.”* (Cannabis clinician 2; 120 medicinal cannabis discussions in the past 6 months, 102 prescriptions).

Some non-cannabis clinicians found cannabis clinics a useful tool for accessing specific information on indications, products, dosage, and regulatory processes. These participants were more comfortable continuing a prescription that had been started by a cannabis clinician or other doctor than initiating one themselves. Four GPs and two specialists had consulted a cannabis clinician for guidance or referred patients to these clinics, illustrating some level of integration of cannabis clinics into the health system:

*“There’s a GP that the only thing they do is prescribe cannabis. When I was having trouble with the chap with the gastrointestinal irritation I contacted them and asked their opinion so I guess I find them useful as a source of knowledge. It’s hard to know where to find the knowledge. So, from my point of view actually they’re quite useful.”* (Specialist 15; 3 medicinal cannabis discussions in the past 6 months, 3 prescriptions).

When asked about their views on the training provided by cannabis clinicians, the majority of participants not working in cannabis clinics were strongly opposed. Most commented on the risks of encouraging premature cannabis prescribing without strong scientific evidence to support their recommendations (vis-à-vis established treatment pathways with clinical trial evidence):

*“Training on anything is always useful, but if you’ve got something that has not really indicated a really good position or a really good reason to use it, all the training in the world isn’t going to change your mind about something that appears to be as yet less effective than the things that we’ve already got.”*(Specialist 14; 12 medicinal cannabis discussions in the past 6 months, 0 prescriptions).

Despite the above scepticism, most participants were willing to further their understanding of MC via training if delivered by a source they deemed credible. Trusted sources included government bodies, researchers, and medical association representatives.

### Physicians’ concerns about cannabis clinics

#### Conflict of interest

GPs and specialists were critical of the dual role some cannabis clinicians occupied as prescribers and suppliers of cannabis-based products. Most respondents objected to health professionals having a commercial stake in the medications they prescribe, in other words promoting the cannabis-based products they sell at their clinics for profit. Some respondents felt the cannabis clinics that operate clinic-affiliated dispensaries charged high prices for their products and that these costs were borne by patients via price paid for products. Cannabis clinics were highlighted as a transactional exchange between patients seeking a legal avenue to procure cannabis for broad medical purposes and the clinicians who provide these products:

*“If your only tool is a hammer, everything looks like a nail, so anybody that comes into that clinic, they will find a reason for selling them their product and you know there will be mark-ups on what they sell...”* (Specialist 10, 12 medicinal cannabis discussions in the past 6 months, 0 prescriptions).

#### Focusing on a single treatment option

Non cannabis-clinic participants commented that there are no other specialist clinics centred on a single medical treatment. This was acknowledged as a marked difference between the treatment options available to patients at cannabis clinics compared to other GP-run practices specialising in a clinical area; the latter having a range of therapies available to them within a clinical sub-specialty. GPs and specialists discussed the risks of developing a medical practice based on a single drug, in particular the opportunity to exploit legal access to cannabis for recreational purposes:

*“There’s no clinic you know we don’t do that for any other medication …*. *it doesn’t make any sense so if you’re using it, if it’s a part of your formulary then go to your GP and they’ll talk about if it’s the right medication for you. But having a clinic that just exists to sell one drug is open for abuse.”* (Specialist 7, 7 medicinal cannabis discussions in the past 6 months, 7 prescriptions).

A handful of participants questioned why cannabis clinicians are willing to prescribe cannabis for any health condition, despite their perception of limited scientific evidence available to support its use. Some respondents raised the issue of bias in the clinical judgement of cannabis clinicians leading to inappropriate recommendations:

*“My only concern is that they are providing only one medicine and matching that to problems rather than the other way around which doesn’t seem like the right flow of logic.”* (GP 6; 4 medicinal cannabis discussions in the past 6 months, 1 prescription).

#### Compartmentalisation of health care

Another key issue put forward was the risk of cannabis clinics compartmentalising patient care by providing a service that could otherwise be delivered by GPs. In some instances, the emergence of cannabis clinics has enabled GPs to bypass learning about cannabis by shifting the responsibility of prescribing to cannabis clinics:

*“I think that has meant that other health professionals or prescribers can kind of go, ‘Well, I don’t know nothing about this, and I don’t need to know anything about this because if you want cannabis, you can go and see the cannabis clinic.’”* (Specialist 8; 25 medicinal cannabis discussions in the past 6 months, 6 prescriptions).

Some interviewees, specifically palliative care specialists, were concerned about patients engaging numerous treatment providers, arguing this can adversely affect continuity of care and create conflicts within treatment plans. They commented that the nature of palliative care already requires patients to engage a network of intertwining physicians and the addition of a service that focuses on a specific issue without addressing the whole can be counterproductive to holistic treatment. One palliative care specialist commented that they would rather upskill in a specific drug than refer patients to a separate provider:

“*Why do we need these cannabis clinics? I don’t think compartmentalisation of care is a good thing. Having lots of different doctors treating different things. That’s been shown to be harmful. You should have one doctor and if you need a specialist for a specific thing, it’s fine but cannabis prescribing is not complicated. I think it’s ridiculous seeing a cannabis specialist. It should just be the GP … Literally, it can take an hour to learn how to prescribe cannabis products and if there’s one GP that doesn’t know, then surely, there’s another GP in the clinic that the patient can see, rather than going to a different clinic.”* (GP 4; 20 medicinal cannabis discussions in the past 6 months, 5 prescriptions).

## Discussion

This research explored the complexity of integrating cannabis clinics into a nation’s health system and employed a qualitative approach to gain in-depth insights into physicians’ views, beliefs, and experiences with MC and cannabis clinics, beyond broad quantitative descriptions. Cannabis clinicians outlined a number of reasons for establishing cannabis clinics in the health system, including providing education for other physicians, building expert knowledge in a niche clinical speciality, and meeting patient demand for MC prescriptions. In contrast, non-cannabis GPs and specialists expressed concerns about conflicts of interest, the limited scientific evidence base for recommending cannabis, and risks to continuity of patient care.

It was acknowledged by some non-cannabis clinicians and described by cannabis clinicians that cannabis clinics provide a non-judgmental environment to provide access to cannabis therapies. Past research has shown that potential stigma is a barrier for patients and physicians considering cannabis as a treatment option [25, 26]. For example, Rychert et al. survey found many patients were concerned they would be judged by their health provider if they made a request for a medicinal cannabis prescription [9]. Another study of NZ general practice patients (*N* = 134) found less than 10% were comfortable discussing medicinal cannabis with a physician or specialist [27]. Research has also shown that physicians’ negative bias and stigma may compromise their consideration of medicinal cannabis as an effective treatment option [28]. The historic status of cannabis as an illegal drug suggests the need for trust in the patient-physician relationship to alleviate patients’ fear of judgement and facilitate an open dialogue [8].

Non-cannabis clinicians identified the issues that challenged the long-term integration of cannabis clinics into the health system as poor scientific evidence to support cannabis therapy, the potential conflict of interest in servicing patient demand, and the risk of compartmentalisation of care when multiple providers are engaged. The high price of cannabis products and cannabis clinic consultations were commented on by a number of participants, raising questions about the equity of access to medicinal cannabis in New Zealand, which should be explored in future research. Other countries have implemented measures in attempts to mitigate the potential for this inequity. For example, in 2017 the German government passed legislation that required health insurance providers to cover payments for pharmaceutical-grade cannabis treatments for patients with a prescription from a physician, subject to conditions, and provide a strong justification in the case of a rejected claim. The implications were that the barrier of cost may lessen for patients who received a reimbursement for their prescription payment, however other issues (i.e., bureaucratic application process, illegal self-cultivation, and physicians’ hesitance to prescribe) persist [29]. In the US, some medical cannabis programmes impose conditions to minimise the risk of inappropriate cannabis recommendations, such as mandatory training in cannabis prescribing, prescriptions with a 30-day maximum threshold, and monitoring of the safety and value of cannabis treatment [30].

This paper adds to other studies that have identified education and training as enablers to delivering more informed medicinal cannabis advice [31–36]. Participants in our study suggested that upskilling clinicians, particularly GPs, in MC therapies through training and resources delivered from trusted and independent sources may encourage patients to engage with their established GPs, who have their medical history. This may reduce the number of medical providers patients need to engage and create opportunity for cannabis clinics to act as a knowledge hub for other health professionals and a specialist service for complex cases. This study also brings novel insight into the role cannabis clinicians feel they play in educating other health professionals and servicing patients in the health system. A few respondents discussed reclassifying medicinal cannabis products as alternative therapies/herbs that could be accessed over-the-counter at pharmacies without prescription and physician consultation, an idea the authors of this paper have also proposed [37]. Some jurisdictions in Australia, Europe, and the United States have already made some non-intoxicating cannabinoid products available to the public without prescription for broad therapeutic purposes [38, 39]. This would disestablish doctors as the gatekeepers to some categories of MC products, simultaneously removing the access barrier caused by reluctance to prescribe, and requiring patients rather than prescribers to assume the responsibility and risk associated with cannabis therapy. There is, however, a risk this approach may result in patients not consulting a physician about a health problem and receiving a formal diagnoses and treatment plan.

In conclusion, while the number of cannabis clinics continue to grow in New Zealand, poor understanding and acceptance of their role in patient care challenge their long-term integration into the health system and the care of patients. Going forward, there is a pressing priority for clinical trial evidence for the efficacy of MC treatments, particularly for commonly prescribed conditions, to mitigate the personal risks and concerns physicians currently feel when prescribing based on clinical-judgement. This would involve investment in randomised controlled trials (RCT) on MC, the “gold standard” for conducting research, to contribute to more informed clinical decision-making and patient safety. Findings from RCT [40] could be supplemented with “real-world evidence” from patients prescribed MC, including data from non-interventional studies, audits of medical records and registries [41, 42]. Australia’s Therapeutic Goods Administration (TGA) also have a recommendation pending that would allow the cultivation, extraction, purification, manufacture and research of cannabis-based products under a single licence, to reduce compliance burden placed on suppliers [43]. The NZMCS may consider such changes to support research and patient access to medicinal cannabis and reduce procedural barriers in the current structure. In the short term, it is recommended that continuing medical education on medicinal cannabis provide succinct and current information on recent scientific studies, dosage, and legal and regulatory changes, and GPs and specialists routinely attend these sessions to upskill their clinical practices [28].

### Strengths and limitations

The recent implementation of New Zealand’s Medicinal Cannabis Scheme means medical practitioners in this study may have less knowledge and experience of cannabis clinics compared to more established regimes overseas. The purposive sampling facilitated interviews with physicians who have had recent experiences of discussing medicinal cannabis in their clinical practice but is not intended to be a representative sample of all physicians in New Zealand. The in-depth qualitative interviewing method elicited a rich understanding of cannabis clinics from the perspectives of cannabis clinicians, general practitioners, and specialists. Recruiting clinicians from diverse backgrounds, including private and public settings in rural and urban locations, and with different levels of seniority and experience contributed to a wide range of views in the data. The findings of this study provide insights for other medicinal cannabis regimes integrating cannabis clinics in their health sectors.

## Data Availability

The larger dataset generated and analysed during the current study are not publicly available due to confidentiality reasons (i.e. contains information that could compromise research participant privacy/consent) but may be available from the corresponding author on reasonable request.
