# Correction: The role of cannabis clinics in the health system: a qualitative study of physicians’ views in New Zealand

**DOI:** 10.1186/s12913-023-09084-5

**Published:** 2023-01-23

**Authors:** Vinuli Withanarachchie, Marta Rychert, Chris Wilkins

**Affiliations:** Shore & Whāriki Research Centre, College of Health, Massey University, Auckland, New Zealand

**Correction: BMC Health Serv Res 23, 10 (2023)**

**https://doi.org/10.1186/s12913-022-09021-y**

Following publication of the original article [1], the authors identified an error in the author names. The given names and family names of all authors were erroneously transposed.

The incorrect author names are: Withanarachchie Vinuli, Rychert Marta and Wilkins Chris

The correct author names are: Vinuli Withanarachchie, Marta Rychert and Chris Wilkins

The author group has been updated above and the original article [1] has been corrected.
